# Impact of Glutathione on Wines Oxidative Stability: A Combined Sensory and Metabolomic Study

**DOI:** 10.3389/fchem.2018.00182

**Published:** 2018-06-08

**Authors:** Maria Nikolantonaki, Perrine Julien, Christian Coelho, Chloé Roullier-Gall, Jordi Ballester, Philippe Schmitt-Kopplin, Régis D. Gougeon

**Affiliations:** ^1^UMR PAM Université de Bourgogne/AgroSup Dijon, Institut Universitaire de la Vigne et du Vin - Jules Guyot, Dijon, France; ^2^Centre des Sciences du Goût et de l'Alimentation, UMR 6265 Centre National de la Recherche Scientifique, UMR 1324 INRA-Université de Bourgogne Franche Comté, Dijon, France; ^3^Research Unit Analytical Bio Geo Chemistry, Helmholtz Zentrum Muenchen, Neuherberg, Germany; ^4^Technische Universität München, Analytical Food Chemistry, Freising, Germany

**Keywords:** chardonnay wine, oxidative stability, FT-ICR-MS, sulfur compounds, peptides

## Abstract

This paper is a comprehensive study regarding the role of glutathione as a natural antioxidant on white wines aging potential. It includes sensory and ultrahigh resolution mass spectrometry (FTICR-MS) metabolomics of aged chardonnay wines from 2008 to 2009 vintages, made after glutathione spiking at alcoholic fermentation or bottling. The closure effect was also considered. The sensory analysis revealed a clear vintage, closure and glutathione effect on wines oxidative character after several years of bottle aging. Spearman rank correlation was applied to link the sensory analysis and the exact mass information from FT-ICR-MS. FTICR–MS along with multivariate statistical analyses put in evidence that glutathione efficiency against wines sensory oxidative stability is related to wines antioxidant metabolome consisting of N- and S- containing compounds like amino acids, aromatic compounds and peptides. The chemical composition and origin of wines antioxidant metabolome suggests that its management since the very beginning of the vinification process is a key factor to estimate wines aging potential.

## Introduction

Glutathione (reduced form of glutathione, GSH) is an essential metabolite with multiple functions in plants, foods and beverages (Vermeulen et al., 2005, 2006). Its roles include working as a source of reductant in many oxidation reactions, protecting against heavy metals toxicity, or lipids and polyphenols oxidation as ROS-scavenger (Dimitrova et al., 2010; Kreitman et al., 2013; Nimse and Pal, 2015). GSH is also reported being an important antioxidant, reacting as nucleophile substance that conjugates straightforwardly with reactive electrophiles resulting in foods and beverages chemical oxidative stability (Cilliers and Singleton, 1990; Nikolantonaki and Waterhouse, 2012; de Almeida et al., 2013; Nikolantonaki et al., 2014; Webber et al., 2014). Particularly in wines, recent studies demonstrated the beneficial influence of the addition of glutathione in the white wine production technology, especially for the preservation of the varietal character and color stability (Roussis et al., 2007; El Hosry et al., 2009; Ugliano et al., 2011). GSH is known to scavenge *o*-quinone compounds efficiently in wine/juice conditions (Cheynier et al., 1988; Nikolantonaki et al., 2014). It can react with hydrogen peroxide and undergo addition reactions with carbonyl compounds, although these reactions have not been studied yet in wine-like conditions (Włodek, 1988). Wine related volatile and non-volatile carbonyl compounds constitute the primary oxidation reaction products in wine acidic conditions, and their reactivity with GSH could suggest an efficient management of the oxidation mechanism.

GSH levels decrease during aging while it is known to exhibit potent protection for important aroma compounds such as esters, mono terpenes and volatile thiols (Lavigne and Dubourdieu, 2002; Roussis et al., 2007; Ugliano et al., 2011). Our research showed that the mechanism for this aroma protection is based on its ability to react as a sacrificial nucleophile with quinones (Nikolantonaki and Waterhouse, 2012). GSH's role in protecting aroma compounds or otherwise inhibiting premature oxidative aging is appreciated by winemakers, but poorly documented or explained. However, it is shown that spiking wine with GSH at bottling, limits the accumulation of acetaldehyde and preserves aromatic complexity and freshness after 12 months of storage (Webber et al., 2017).

In this study, we have investigated the effects of GSH addition after alcoholic fermentation and/or prior to bottling on the development of Chardonnay wines oxidative character during bottle aging. Trials were carried on with Chardonnay wines from two consecutive vintages, bottled with synthetic closures or screw caps. Synthetic closures were selected due to their consistent high OTR across replicate closures. Here, for the first time sensory analysis and untargeted molecular analyses by Fourier transform ion cyclotron resonance mass spectrometry (FT-ICR-MS) were combined for unraveling the complex and long-lasting chemical modifications induced in wine matrices after GSH spiking in relation to their oxidative stability.

## Materials and methods

### Wine trials

Chardonnay wines from Burgundy were produced from grapes originating from the same vineyard during 2008 and 2009 vintages, following the same winemaking process at the same winery. GSH (Sigma Aldrich, France) spiking was done after alcoholic fermentation at 50 mg/L (50AF thereafter) or prior bottling at 20 and 50 mg/L (20B and 50B, respectively thereafter) and compared to the control wines with no GSH spiking. Wines were conditioned in standard commercial 75 cl green glass bottles and stored at 16°C until analysis. In order to enhance differences in oxygen exposure, samples were sealed either with synthetic coextruded stoppers (S) or screw caps (C) allowing high and low oxygen ingress, respectively during aging. Wines were analyzed in April 2016 (i.e., after 7 and 6 years of bottle aging) in biological duplicates.

### Sensory analysis

A trained panel of 16 oenology students from the University of Burgundy (four females and 12 males, average age 24.2 years old; *SD* = 1.2) took part in this study. These panelists had been extensively trained for 2 months specifically on the wine oxidation and reduction aromas. 15 out of 16 students were qualified for the evaluation session. Sensory analyses took place in a sensory room equipped with individual booths. Samples of wine were assessed in standardized black glasses (opaque) coded with 3-digit numbers. Each sample contained 30 mL of wine at room temperature. First, participants were asked to rate the intensity of oxidation-reduction (called here REDOX-sensory score) of the samples orthonasally (i.e., by smell only) and afterwards globally (i.e., nose and palate) using 11-point discrete scale according to the protocol proposed by Ballester et al. (2018). The scale was anchored between −5 = reduced and + 5 = oxidized, with 0 = neither reduced nor oxidized. For both sessions, the order within the eight wines was specific for each participant and followed a William's Latin square.

### FTICR-MS analysis

Sample preparation and analysis was carried out as described by Roullier-Gall et al. (2014b). Freshly opened wine bottles were sampled and diluted in a 1:50 (v/v) ratio in pure methanol. No extraction or purification steps were performed. Samples were measured randomized immediately after dilution. The samples were directly infused on a Bruker solariX Ion Cyclotron Resonance Fourier Transform Mass Spectrometer (FT-ICR-MS) (BrukerDaltonics GmbH, Bremen, Germany) equipped with a 12 Tesla superconducting magnet (Magnex Scientific Inc., Yarnton, GB) and a APOLO II ESI source (BrukerDaltonics GmbH, Bremen, Germany) operated in the negative ionization mode. The diluted wine samples were loaded onto an autosampler with a volume of 2 μl.min^−1^. As for previous studies mass spectra were acquired with a time domain of 4 mega words over a mass range of *m/z* 92–1,000 and 300 scans were accumulated per sample (Gougeon et al., 2009; Roullier-Gall et al., 2014b). Spectra were externally calibrated daily on clusters of arginine (10 mgL^−1^ in methanol).

### Data processing

Raw spectra were post-processed by Compass Data Analysis 4.2 (Bruker Daltonics, Bremen, Germany). Spectra were internally recalibrated with a reference list including fatty acids and recurrent wine compounds up to *m/z* 800, with mass errors below 50 ppb. Peaks with a signal-to-noise ratio (S/N) of at least 6 were exported to mass lists as ASC files. All exported *m/z* features were aligned (peak alignment window up to 1 ppm) in a matrix containing average *m/z* values and corresponding peak intensities, using an in-house developed software. The aligned data matrix was filtered by mass signals count (masses common to < 2 samples were removed) and mass defect above 0.8. Molecular formulas were assigned to *m/z* values by mass difference network analysis using an in-house developed software tool Netcalc (Tziotis et al., 2011) with network tolerance: 0.2 ppm and Net Calc tolerance: 0.2 ppm. Generated formulas were validated by setting plausible chemical constraints, including isotopic pattern search, N rule, O/C ratio ≤ 1, H/C ratio ≤ 2, element counts: C ≤ 100, H ≤ 200, O ≤ 80, N ≤ 3, S ≤ 3, and P ≤ 1.

### Data mining

Principal Component Analysis (PCA) and Hierarchical Cluster Analysis (HCA) were obtained with Perseus 1.5.1.6 (http://www.perseus-framework.org, Max Planck Institute of Biochemistry, Germany) (Tyanova et al., 2016). The clustering was performed using a Pearson's correlation. Spearman rank correlations were performed in MS Excel 2010 (Microsoft, Redmond, USA). The rank correlation between REDOX-sensory scores and FT-ICR-MS intensities rankings was computed for each mass and for each component according to Spearman correlation. The total procedure is describe by Herzsprung et al. (2012).

## Results and discussions

### Wines sensory evaluation

Orthonasal and global sensory analysis was undertaken to evaluate the oxidative and reductive state of wines in a scale (+5 < −5) by 15 trained panelists. First, a three-way ANOVA with vintages (2008 and 2009), closures (synthetic corc (S) or screw cap (C) and GSH treatment (Control; 50_AF; 20_B; 50_B) as within subject factors, was carried out for both orthonasal and global evaluations. The interactions included in the model were vintage^*^closure, vintage^*^GHS, closure^*^GHS and vintage^*^closure^*^GHS. With respect to the orthonasal data, vintage and closure were the major effects, showing significant differences (*F* = 8.1, *p* = 0.005 for vintage and *F* = 195.5, *p* < 0.0001 for closure). The interaction vintage^*^closure was also significant (*F* = 9.55, *p* = 0.002). With respect to the global evaluation data, the ANOVA showed the same significant effects: vintage (*F* = 8.17, *p* = 0.005), closure (*F* = 131.4, *p* < 0.0001) and the interaction vintage^*^closure (*F* = 8.14, *p* = 0.005).

**Figure 1:**
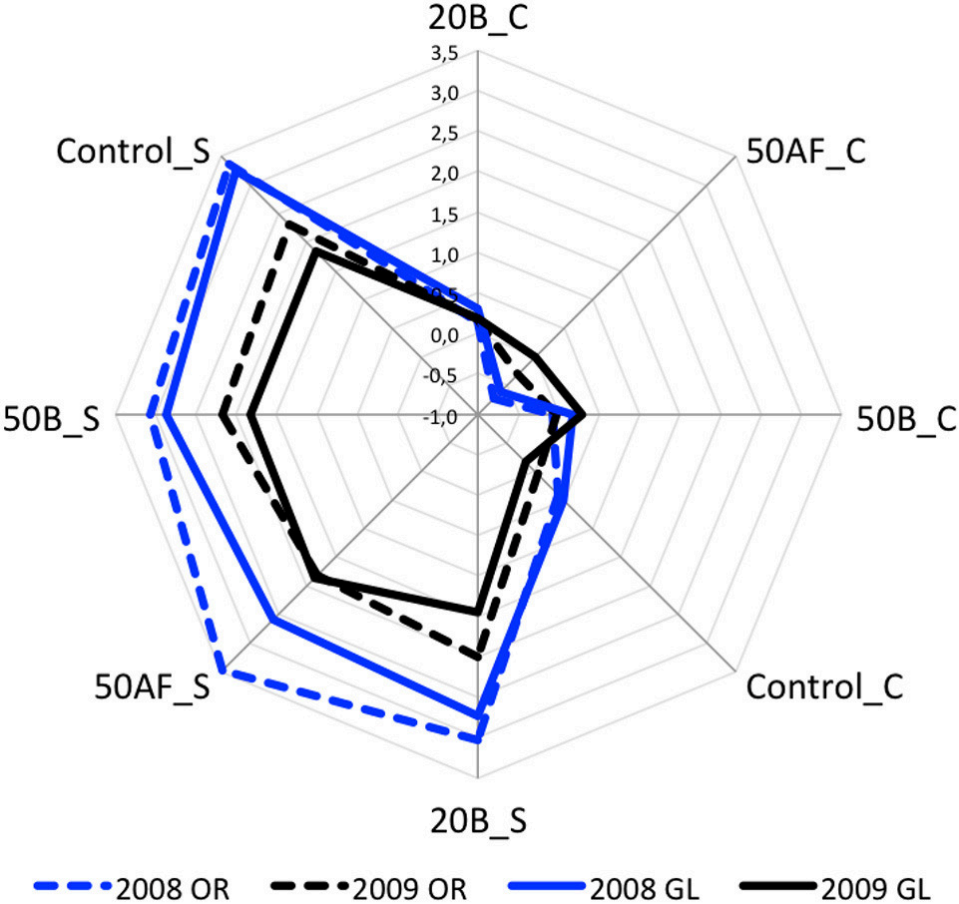
Average global (GL) and orthonasal (OR) REDOX-sensory scores for wines from 2008 (in blue) to 2009 (in black) vintages, treated with GSH after alcoholic fermentation (50AF) or at bottling (20B; 50B) and bottled with screw cap (C) or synthetic cork (S).

Figure 1 shows the average REDOX-sensory scores for each sample, each vintage and each evaluation condition. This figure illustrates the main significant effects as well as the interactions between tested variables. Orthonasal and global sensory analysis for 2008 and 2009 vintages demonstrated that screw cap wines (C) had no oxidative evolution compared to synthetic cork (S) ones. Given the lack of variability among screw cap wines for both vintages, the further discussion was based only to synthetic cork wine samples. Synthetic cork having higher OTR presented important evolutions in wine matrices in a vintage dependent manner. Figure 1 shows also a clear vintage effect for S closures, with 2008 being less resistant to oxidation than 2009 whatever the GSH treatment considered. The relative low oxidative evolution of burgundy chardonnay wines from 2009 vintage was also recently reported indicating the importance of vintage effect on wines aging potential during bottle aging (Coelho et al., 2018). In our experiment, the vintage effect was not noticeable for C closures. Finally, the two sensory evaluation conditions followed similar patterns. Given the strong vintage effect, we decided to carry out an ANOVA for each vintage separately (Table 1). All ANOVAs showed a significant closure effect and non-significant closure^*^GSH interactions. More interestingly, the ANOVA of the 2008 for global assessment showed also a GSH effect.

**Table 1:** Results of the ANOVAs for each vintage and each sensory evaluation condition.

	**2008 OR**		**2008 GL**		**2009 OR**		**2009 GL**	
	**F**	**Pr > F**	**F**	**Pr > F**	**F**	**Pr > F**	**F**	**Pr > F**
PANELIST	1.165	0.31	1.773	0.048	1.408	0.164	1.822	0.046
CLOSURE	190.503	<**0.0001**	157.234	<**0.0001**	50.064	<**0.0001**	29.894	<**0.0001**
GSH	0.849	0.47	2.81	**0.043**	0.383	0.766	0.124	0.946
CLOSURE^*^GSH	1.61	0.192	0.559	0.643	0.109	0.955	0.298	0.826

*Significant effects are presented in bold characters*.

In order to have a closer look at the GSH effect, the Newman-Keuls *post-hoc* comparison for redox scores of GSH means was conducted. Among 2008 GSH treatments, the 50AF exhibited the significantly lowest redox score, followed by 20B, 50B and then by the control. The GSH treatment in 2008 thus appeared to be more efficient when applied at the early stages of the winemaking process (50AF). However, no significant sensory effect of the GSH treatment was found for 2009 samples, which could likely be due to the particular composition of the 2009 wine matrix in terms of resistance to oxidative processes. Taken together, these results clearly highlighted the yet unraveled contribution of wine matrices to the efficiency of GSH treatments, therefore indicating the importance of untargeted metabolomics approach for a more exhaustive characterization of wines complex chemical diversity that could be related to their sensory oxidative evolution.

### Wines chemical diversity evolution during aging

**Figure 2:**
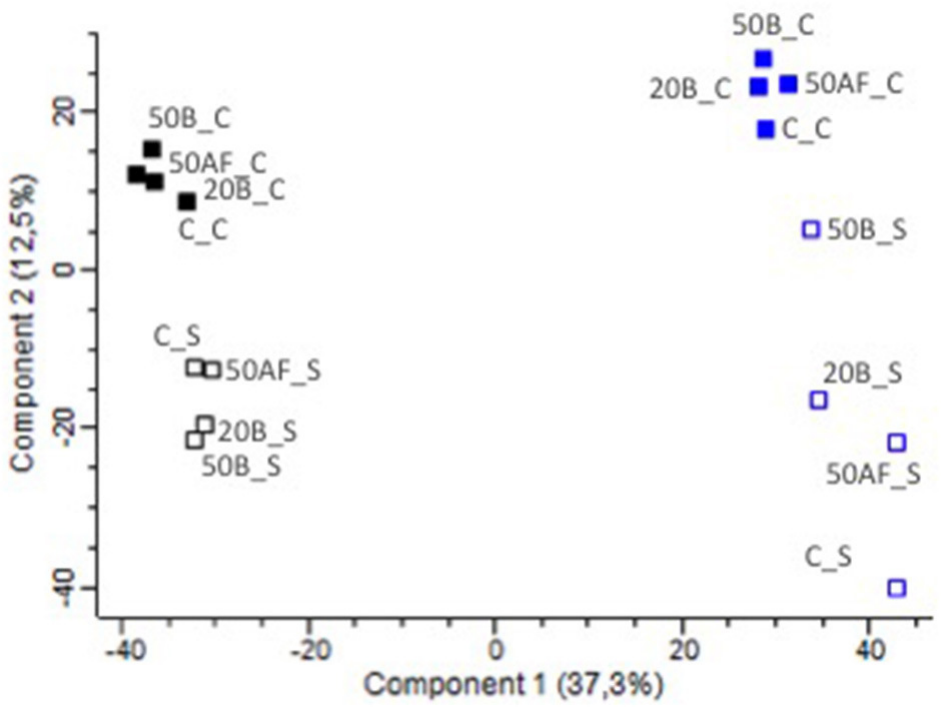
PCA plot of untargeted FT-ICR-MS analysis in negative mode of wines from 2008 (in blue) to 2009 (in black) vintages, treated with GSH after alcoholic fermentation (50AF) or at bottling (20B; 50B) and bottled with screwcap (C) or synthetic cork (S).

Molecular profiling by Fourier transform ion cyclotron resonance mass spectrometry (FT-ICR-MS) enables a comprehensive description of the wine metabolome (Roullier-Gall et al., 2014b). Full dataset multivariate unsupervised statistical analysis through PCA of the ~4,000 annotated elementary formulas are presented in Figure 2. It shows the remarkable and straightforward discrimination of both vintages along the first axis and types of closure along the second axis, revealing the higher impact of these two parameters on the chemical diversity of our bottle aged wines (Roullier-Gall et al., 2014a, 2016). Screw cap samples (C) appeared well grouped compared to synthetic cork (S) samples, for the 2008 and 2009 vintages, indicating specific chemical signatures associated with distinct oxygenation states of wines during aging, within each vintage (Table S1). However, unsupervised multivariate analysis on the whole dataset could not clearly distinguish samples according to GSH treatments. This indicates that discriminating the GSH wine treatment effect is a challenging task, which must be addressed with a specific data mining strategy as developed later in the paper.

### Correlation of wines sensory analysis and chemical diversity related to FT-ICR-MS derived molecular formulae

**Figure 3:**
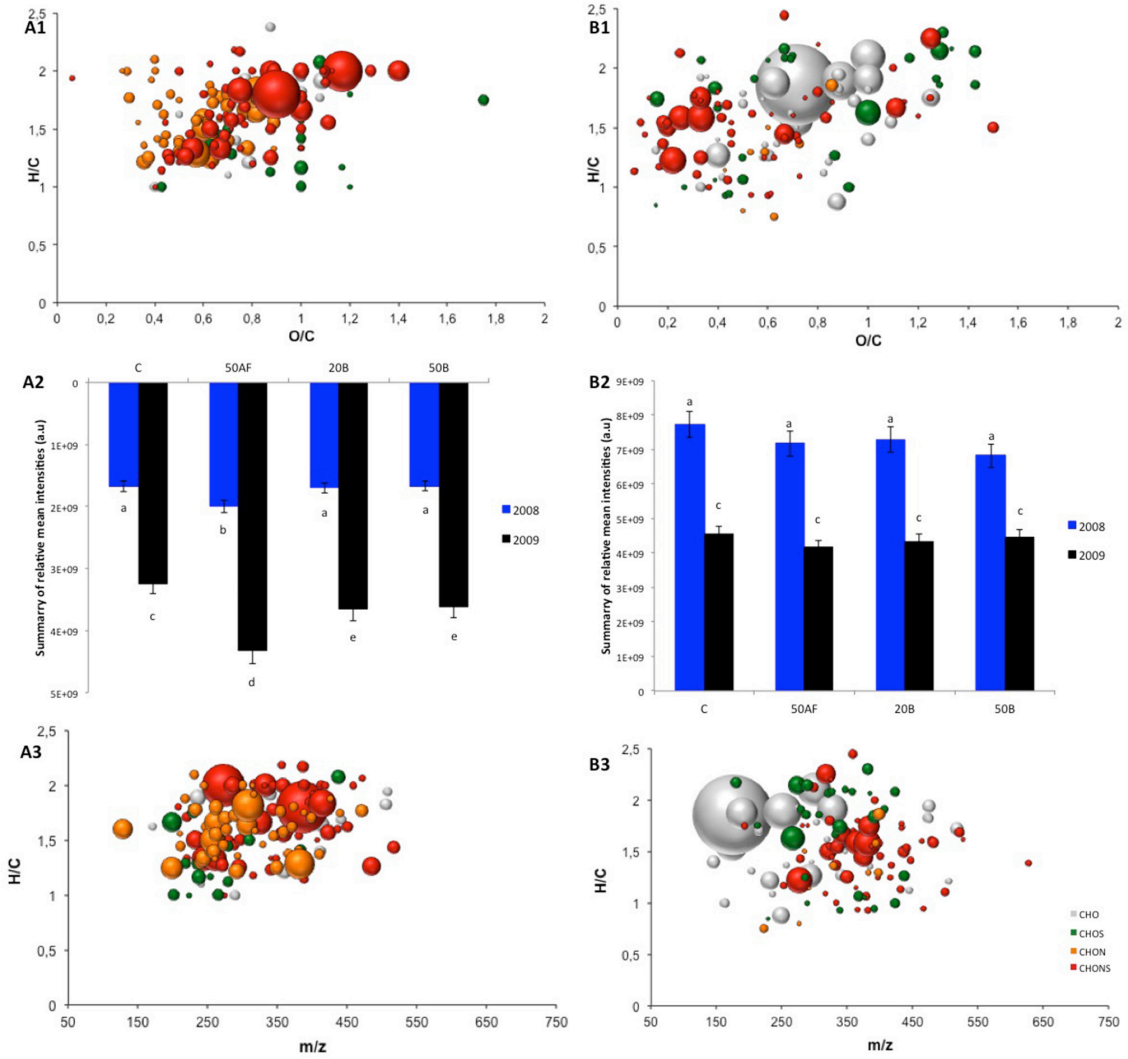
Van Krevelen diagram of assigned molecular formula obtained by FT-ICR-MS with negative **(A1,A3)** and positive **(B1,B3)** correlations with the orthonasal REDOX-sensory scores of wines from 2008 to 2009 vintages, treated with GSH after alcoholic fermentation (50AF) or at bottling (20B; 50B), and bottled with synthetic corks. The significance threshold for Spearman rank correlation was set to 0.604 (α = 0.001). Van Krevelen plots were colored according to molecular classes, i.e., CHO (gray), CHON (orange), CHOS (green), CHONS (red). Bubble sizes correspond to the relative intensities of mass peaks obtained by FT-ICR-MS. Distribution of the summary of relative mean intensities of assigned molecular formulas negative **(A2)** and positive **(B2)** correlated with the orthonasal REDOX scores.

We explored the covariability of each one of 3954 molecular formulae identified from the FT-ICR-MS analysis of wines from 2008 to 2009 with the sensory orthonasal REDOX-sensory scores intensities by means of Spearman's rank correlations (±0.604 confidence limit). Our statistical analysis indicated that 3.7 and 7.6% of the 3,954 assigned molecular formulae were positively and negatively correlated, respectively, with the sensory orthonasal REDOX-sensory scores. 171 negative and 199 positive correlated molecular markers were presented in a van Krevelen diagram, which is a graphical plot of elemental H/C ratios against either elemental O/C rations (Figures 3A1,B1 for negative and positive correlation, respectively) or *m/z* values (Figures 3A3,B3 for negative and positive correlation, respectively). Elemental formulas negatively correlated to orthonasal REDOX-sensory scores, i.e., which exhibited decreasing relative intensities for increasing REDOX-sensory scores, and thus would be markers of oxidative stability, include mainly CHONS formulas (65%), while other series made up by minor fractions: CHON (16%), CHOS (11%), and CHO (8%) (Figures 3A1,A3). The dominance of CHONS and CHOS molecular series indicates the importance of N and S-containing compounds to the sensory related oxidative stability of wines upon bottle aging. Based on H/C and O/C ratios, CHOS and CHONS formulae appeared in the amino sugars region (H/C = 1.5–2.2, O/C = 0.3–0.67), and in the condensed aromatics region (H/C = 0.5–1.5, O/C = 0.67–1.2) (Gougeon et al., 2009; Lu et al., 2016). A large majority (85–98%) of sulfur-containing formulas had O/S values > 4, suggesting that these formulas could correspond to sulfonation. Furthermore, the corresponding *m/z* distribution of CHONS and CHOS molecular markers comprised in a mass range up to 550 *m/z*, demonstrated the importance of low molecular weight N, S containing amino acids, aromatic compounds and peptides on wines sensory oxidative stability during bottle aging. As examples, some *m/z* ions negatively correlated with orthonasal REDOX-sensory scores, were assigned to C_3_H_7_O_5_NS_2_, C_7_H_11_O_5_NS, and C_8_H_15_O_9_NS_2_ elemental formulas, which could be hypothetically assigned to cysteinesulfonic acid, N-acetyl carbocysteine and glucocapparin, respectively. However, we did not undertake fragmentation experiments to confirm the suggested structures, due to low relative intensity signals but further characterization will be done in the future. On the other hand, we considered that tracer compounds positively correlated with orthonasal REDOX-sensory scores were markers of wines sensory instability. Elemental formulas associated with CHONS (42%), CHO (26%), CHOS (25%), and CHON (7%) molecules below 650 *m/z* were assigned with the highest positive correlation scores (Figures 3B1,B3). Elemental formulas annotated as C_9_H_8_O_3_, C_7_H_10_O_5_, C_12_H_22_O_4_, C_12_H_14_O_7_, C_14_H_24_O_5_, C_15_H_18_O_9_, and C_13_H_16_O_2_ were putatively annotated as hydroxycinnamic acid, shikimic acid, dodecanoic acid, phenyl glucuronide, hydroxytetradecanedioic acid, caffeic acid glucoside and isobutyl cinnamate, revealing a direct incidence of phenolic and fatty acids onto the genesis of white wine oxidative character during aging.

The distribution over GSH treatments of the sum of relative mean intensities of all signals negatively correlated with orthonasal REDOX-sensory scores, shown clearly the impact of the vintage on the abundance of wines intrinsic compounds related to the global matrix property to resist against oxidation (Figure 3A2). Wines from 2008, which presented more pronounced REDOX-sensory scores than those from 2009, were on average two times less concentrated in molecular markers associated with their sensory related antioxidant stability during bottle aging. These molecular markers could be considered as part of an antioxidant metabolome, i.e., a pool of intrinsic low molecular weight compounds—most of them yet unknown—collectively contributing to the oxidative stability of Chardonnay wines that could be used in the future to characterize the wines potential ageability. In addition, the GSH addition at the early stages of the vinification process (50AF), led to a significantly (*p* < 0.01) higher proportion of the antioxidant metabolome compared to GSH late additions (whatever the amount added, 20B and 50B), and regardless of the vintage. In contrast, the distribution of the sum of relative mean intensities of all signals positively correlated with orthonasal REDOX-sensory scores was significantly extendable amongst vintages and was not impacted by the GSH spiking (Figure 3B2). The concentration of these compounds dropped considerably from the high values seen in 2008 to 40% in 2009 wines, where the sensory oxidative evolution was less expressed during bottle aging, consistently with the above results.

These results suggest that the antioxidant metabolome of white wines should be highly dependent to the management of N- S-containing compounds since the very beginning of the winemaking process, while compounds related to grape phenolic maturity and pre-fermentive processes dominated by the vintage effect are related to wines oxidative evolution during bottle aging. Future work is needed to address the respective role of the different “*terroirs*,” cultural and winemaking practices and their interactions in the context of wines antioxidant metabolome modifications in order to guarantee a better management of wines potential ageability.

### Mass spectrometric characterization of GSH impact on wines chemical space

**Figure 4:**
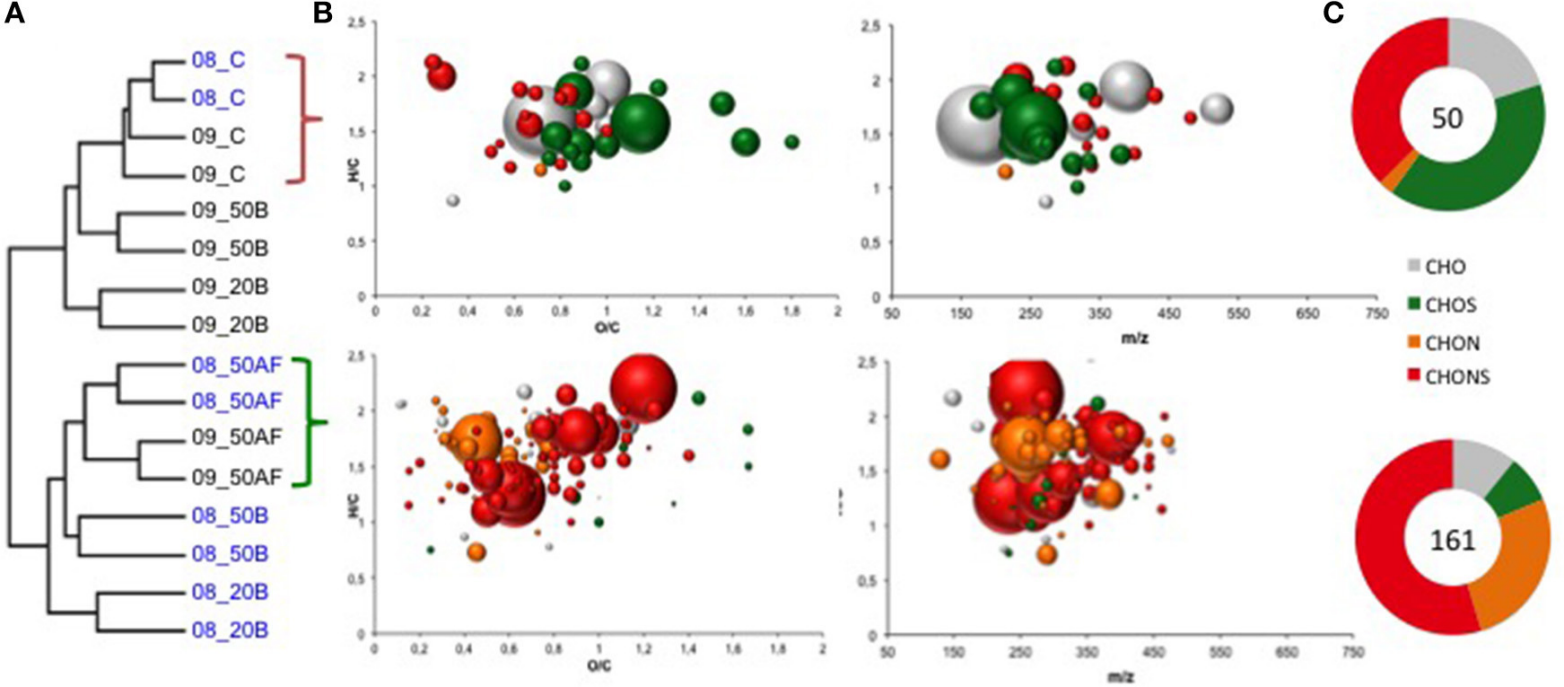
Dataset containing all wine compounds detected by FT-ICR-MS that show a significant change in intensity related to the GSH treatment. **(A)** Hierarchical cluster analysis of the assigned (–)ESI FT-ICR-MS derived molecular formulas observed in wines from 2008 to 2009 treated with GSH after alcoholic fermentation (50AF) or at bottling (20B; 50B) and bottled with synthetic cork. **(B)** Van Krevelen diagrams depict the most representative characteristic molecular formulas for the two main subclusters. Van Krevelen plots were colored according to molecular classes, i.e., CHO (gray), CHON (orange), CHOS (green), CHONS (red). The bubble area depicts the relative mass peak intensity within the respective subcluster. **(C)** Circle histograms indicating the number of molecular formulae presented in the Van Krevelen diagrams.

Hierarchical cluster analysis was performed to group the samples according to their resemblance and to identify the major differences in the molecular wine composition according to the GSH treatments. The analysis revealed two distinct subclusters (Figure 4). The cluster formation resembles in the broadest sense the GSH treatments during the vinification process for both vintages. Regardless of the vintage, the GSH treatment just after alcoholic fermentation (09_AF and 08_AF) resulted a characteristic discriminant chemical modification in the matrix which had a direct effect on wines sensory stability at 7–8 years of bottle aging (Figure 4). The extensive chemical dissimilarity between the subclusters is visible from the ring charts (Figure 4) depicting the distribution of elemental compositions (CHO, CHON, CHOS, and CHONS), along with van Krevelen and *m/z*-resolved H/C atomic ratios of the (-)ESI FT-ICR-MS derived molecular formulas (verified by ANOVA test and *p* ≤ 0.05). The van Krevelen diagrams and ring charts display the most representative molecular formulas for each subcluster and show distinct compositional variations. Up to 150 *m/z* ions exhibited an increasing intensity together with the addition of GSH after alcoholic fermentation. The addition of the linear tripeptide (glu-cys-gly) GSH at the early stages of the vinification process corresponded to the respective prevailing domination of CHONS organic molecules with a contribution to the total intensity of the assigned molecular markers of up to 67%, followed by the CHON (23%) and the CHOS (10%). The CHONS and CHOS most abundant molecular markers for GSH addition had a mass range between 128 and 475 Da and occupied essentially the chemical space of amino acids, peptides, amino sugars and carbohydrates of the van Krevelen diagram, illustrating the importance of N and S-metabolome on wines chemical stability during bottle aging.

### Identification of GSH molecular markers

The next task we addressed is the putative identification of the molecular markers derived after the addition of GSH in wines. As a first step in the analysis of the MS data we searched for metabolites listed in know databases (including KEGG, HMDB, LipidMap and our in house wine and plant database). Twenty nine out of the total 161 chemical formulae GSH markers could be identified putatively and the accuracy of the annotated masses varied between 0.01 and 0.6 ppm (Table 2). The listed compounds include prominent low-molecular-weight organic acids, thiols, amino compounds, peptides and sulfonated compounds already identified in wine or other food matrices. As an example, the ion *m/z* 129.04259 was identified as pyroglutamic acid (Figure S1). This organic acid has been identified in wines as a secondary metabolite of *Saccharomyces cerevisiae* fermenting activity (Pfeiffer and König, 2009) and characterized as a precursor of ethyl 2-hydroxyglutarate, reminiscent of oxidative sensory like burnt caramel, honey and balsamic notes (Schneider et al., 1998). In our experiment, by comparing the relative intensities among samples, pyroglutamic acid (Figure S2) intesity was found to be lower in control wines from 2008 to 2009 than in wines treated with GSH after alcoholic fermentation (50AF) or at bottling (20B; 50B). Figure S2 also shows on average lower pyroglutamic acid intensity in wines from 2008 than 2009 vintage, consistently with above results. Among vintages we observe a loss of 34 and 19% for control wines if we compare with 50AF; 50B and 50AF wines from 2008 to 2009, respectively. The loss of pyroglutamic acid intensity follows the same trend as their oxidative character, suggesting that pyroglutamic acid could contribute to wines sensory instability during bottle aging.

**Table 2:** Assigned GSH molecular markers detected in wines (<0.6 ppm).

**Tentative ID**	**Detected mass [M – H]^−^**	**Theoretical mass**	**error (ppm)**	**Formula**	**Citations**
Pyroglutamic acid	128.03531	129.04259	0.02108	C_5_H_7_O_3_N	Pfeiffer and König, 2009^*^
Mevalonic acid	147.06626	148.07356	0.10403	C_6_H_12_O_4_	Hock et al., 1984
L-Tyrosine	180.06660	181.07389	0.04998	C_9_H_11_O_3_N	Bauza et al., 1995
2-oxo capric acid	185.11816	186.12559	0.43838	C_10_H_18_O_3_	Honda et al., 2015^**^
Cysteine-S-sulfate	199.96915	200.97656	0.49460	C_3_H_7_O_5_NS_2_	Arapitsas et al., 2016
N-heptanoyl-homoserine lactone	212.12909	213.13649	0.57276	C_11_H_19_O_3_N	
O-Succinyl-L-homoserine	218.06690	219.07428	0.48929	C_8_H_13_O_6_N	Knoll et al., 2011
LeuVa	229.15568	230.16304	0.34823	C_11_H_22_O_3_N_2_	Peptides
Leu Asp	245.11424	246.12157	0.21989	C_10_H_18_O_5_N_2_	Peptides
Palmitic acid	255.23293	256.24023	0.09207	C_16_H_32_O_2_	Yunoki et al., 2004
Gamma-L-Glutamyl-L-pipecolic acid	257.11428	258.12157	0.03578	C_11_H_18_O_5_N_2_	Dardenne et al., 1974
Asp Glu	261.07279	262.08010	0.12372	C_9_H_14_O_7_N_2_	Peptides
SerThrGly	262.10446	263.11173	0.00190	C_9_H_17_O_6_N_3_	Peptides
Ser-Gly-OH	283.05719	284.06445	0.07454	C_11_H_12_O_7_N_2_	Peptides
Stearic acid	283.26427	284.27153	0.06354	C_18_H_36_O_2_	Yunoki et al., 2004
Val GlyLeu	286.17723	287.18450	0.01956	C_13_H_25_O_4_N_3_	Peptides
Dihydrokaempferol	287.05612	288.0633	0.02368	C_15_H_12_O_6_	Baderschneider and Winterhalter, 2001
SerAlaOH	297.07278	298.08010	0.13969	C_12_H_14_O_7_N_2_	Peptide
GlyGluCys	306.07650	307.08380	0.10160	C_10_H_17_O_6_N_3_S	Peptide
Epithienamycin B	311.07074	312.07799	0.08615	C_13_H_16_O_5_N_2_S	
Pro Thr Pro	312.15647	313.16377	0.05638	C_14_H_23_O_5_N_3_	Peptide
Asn Asp Ala	317.11032	318.11755	0.15357	C_11_H_18_O_7_N_4_	Peptide
SerGluSer	320.10989	321.11721	0.12995	C_11_H_19_O_8_N_3_	Peptide
Glucocapparin	332.01159	333.01882	0.14909	C_8_H_15_O_9_NS_2_	Gueye et al., 2013^**^
GlyPhe Asp	336.12008	337.12738	0.07884	C_15_H_19_O_6_H_3_	Peptides
Veratric acid glucuronide	357.08269	358.09000	0.09381	C_15_H_18_O_10_	Guillén et al., 1993
Glutathione-S-sulfate	386.03330	387.04000	0.00600	C_10_H_17_O_9_N_3_S_2_	Arapitsas et al., 2016
1-O-Caffeoyl-(b-D-glucose 6-O-sulfate)	421.04454	422.05190	0.18026	C_15_H_18_O_12_S	Flamini, 2013
S-(4-Nitrobenzyl)glutathione/adduct methylnicotinate	441.10870	442.11583	0.32282	C_17_H_22_O_8_N_4_S	Rodríguez-Bencomo et al., 2014

* Identified in wine;

** *Identified in other foods*.

Among the identified putative thiols, we found the sulfonated products of cysteine and glutathione (Figures S3, S5). Both, cysteine-S-sulfite and glutathione-S-sulfite were detected in higher concentrations in the 50_AF wine samples from 2009 compare to the 2008 one (Figures S4, S6). Recently Arapitsas et al. (2016) reported cysteine-S-sulfite and glutathione-S-sulfite in wines bottled and stored under oxidative conditions indicating that the sulfonation mechanism can occur during aging. Under our experimental conditions, the levels of cysteine and glutathione sulfonation were clearly dependent to the vintage and the reductive vinification conditions related to the early addition of GSH during the process. No closure effect was observed for both vintages. Referring to these findings, we suggest that the sulfonation of thiols could occur at the early stages of the vinification process and the sulfonated products remain stable during bottle aging whatever the oxygen intake. However, the characterization of the antioxidant capacity of sulfonated products remains to be determined in order to elucidate the importance of sulfonation on wines oxidative stability.

Another group of markers included a series of small peptides, specifically dipeptides and tripeptides; tentatively identified as leu/val; leu/asp; asp/glu; ser/thr/gly; ser/gly-OH; val/gly/leu; ser/ala-OH; gly/glu/cys; pro/thr/pro; asn/asp/ala; ser/glu/ser; gly/phe/asp. Because of their low concentration in the sample, it was not possible to confirm their sequence. The antioxidant properties of small peptides, generally found in fermenting food, have been reported (Elias et al., 2008; Samaranayaka and Li-Chan, 2011). Antioxidant peptides are generally short peptides (2–10 amino acid residues), and the amino acid sequence is a determinant factor for their efficiency. The presence of certain amino acid residues, notably his, tyr, trp, met, cys and pro is significantly correlated with peptides radical quenching activity (Saito et al., 2003). In the present study, no particular trend was observed in detected peptides relative abundances among GSH treatments. However, a significant vintage effect (*p* < 0.005) was noticed when comparing peptides relative intensities (Figures S7, S8). Wines from the 2008 vintage appeared less concentrated in peptides than those from 2009 when there relative intensities were compared. The vintage effect was even stronger when comparison was based only on pro/thr/pro and gly/glu/cys peptides (Figure S9). These peptides contain amino acids (cys and pro) that could be involved in the antioxidant properties and their presence could partially explain 2009 wines lower oxidative evolution. Considering this results, the antioxidant stability of wines that has been linked to the presence of GSH could also be attributable to other types of small peptides that could have even greater antioxidant properties than GSH.

## Conclusions

This work provides the first combined metabolomics and sensory analysis describing the effect of GSH addition at early or late stages of the vinification process on chardonnay wines chemical diversity and oxidative stability during bottle aging. It brings also important information on the consequence of vintage, closure and time of GSH addition in the context of Chardonnay wines oxidative character evolution. Closure and vintage effects dominated in any case the GSH effect. The sensory evaluation of wines indicated, regardless of the GSH treatment and the vintage, that screw capped wines had no oxidative evolution in contrast with synthetic corked ones. However, based on the comparison of samples with synthetic cork, GSH treatment impacted the oxidative stability of wines in a vintage dependent manner and put in evidence the intrinsic capacity of wine matrices to resist against oxidation. The innovative application of FT-ICR-MS based metabolomics in correlation with the sensory analysis showed the great importance of nitrogen and sulfur containing compounds to wines oxidative stability. Specifically, 21 prominent low-molecular-weight organic acids, thiols, amino compounds, peptides, and sulfonated compounds were annotated, identified putatively and designated as molecular markers of wines oxidative stability and GSH treatment. However, the functional characterization of some unidentified putative candidates is in progress.

## Author contribution

MN, RG and PS-K conceived and designed the experiments. MN, PJ, and CC performed the experiments. MN, PJ, CR-G, JB, and CC analyzed the data. PS-K and RG contributed reagents, materials, analysis tools. MN, JB, PS-K, and RG wrote the paper.

### Conflict of interest statement

The authors declare that the research was conducted in the absence of any commercial or financial relationships that could be construed as a potential conflict of interest.
