# Supplementary material for: Impact of Glutathione on Wines Oxidative Stability: A Combined Sensory and Metabolomic Study
Source: Front Chem. 2018 Jun 8;6:182. doi: 10.3389/fchem.2018.00182 (PMC6002495; doi:10.3389/fchem.2018.00182)
Supplement: Supplementary file 1 [file Presentation_1.PDF]

Supplementary data

Impact of glutathione on wines oxidative stability: a combined sensory and metabolomic study

Maria Nikolantonaki[1]*, Perrine Julien[2], Christian Coelho[1], Chloé Roullier-Gall[1,3,4], Jordi Ballester[2], Philippe Schmitt-Kopplin[3,4] and Régis D. Gougeon[1]

[1]UMR PAM Université de Bourgogne/AgroSup Dijon, Institut Universitaire de la Vigne et du Vin, Jules Guyot, Rue Claude Ladrey, BP 27877, 21078 Dijon Cedex, France

[2]Centre des Sciences du Goût et de l'Alimentation, UMR 6265 CNRS, UMR 1324 INRA-Université de Bourgogne Franche Comté, 9 E Boulevard Jeanne d'Arc, F-21000 Dijon, France

[3]Research Unit Analytical Bio Geo Chemistry, Helmholtz Zentrum Muenchen, 85764 Neuherberg, Germany

[4]Technische Universität München, Analytical Food Chemistry, Alte Akademie 10, 85354 Freising, Germany

Corresponding author: *maria.nikolantonaki@u-bourgogne.fr

Table S1: Discriminant m/z ions according to wine closure (screw cap/ synthetic cork)

	m/z	Theoretical	H	C	O	N	S	P
Screw Cap	193.054	194.061	14	7	4	0	1	0
Screw Cap	310.948	311.955	9	4	12	0	1	1
Screw Cap	389.058	390.065	22	12	10	0	2	0
Screw Cap	310.105	311.112	17	13	6	3	0	0
Screw Cap	351.039	352.046	16	12	10	0	1	0
Screw Cap	375.079	376.086	24	12	9	0	2	0
Screw Cap	306.171	307.178	25	17	4	1	0	0
Screw Cap	467.108	468.115	28	14	15	0	1	0
Screw Cap	303.039	304.046	16	8	10	0	1	0
Screw Cap	405.071	406.078	22	12	13	0	1	0
Screw Cap	301.060	302.067	18	9	9	0	1	0
Screw Cap	328.050	329.057	15	13	7	1	1	0
Screw Cap	207.033	208.041	12	7	5	0	1	0
Screw Cap	307.070	308.078	16	11	6	2	0	0
Screw Cap	234.992	235.999	8	7	7	0	1	0
Screw Cap	543.052	544.059	17	17	13	6	0	1
Screw Cap	423.039	424.046	16	18	10	0	1	0
Screw Cap	277.002	278.010	10	9	8	0	1	0
Screw Cap	410.045	411.052	22	11	9	1	1	1
Screw Cap	311.985	312.993	11	8	8	1	2	0
Screw Cap	340.017	341.024	15	10	8	1	2	0
Screw Cap	229.012	230.019	11	5	8	0	0	1
Screw Cap	260.006	261.013	11	9	4	1	2	0
Screw Cap	373.017	374.024	14	13	7	2	2	0
Screw Cap	380.962	381.969	11	10	8	2	2	1
Screw Cap	326.100	327.107	17	13	7	3	0	0
Screw Cap	391.028	392.035	16	13	8	2	2	0
Screw Cap	385.023	386.031	14	15	10	0	1	0
Screw Cap	325.096	326.104	22	12	8	0	1	0
Screw Cap	315.127	316.134	24	15	5	0	1	0
Screw Cap	179.038	180.046	12	6	4	0	1	0
Screw Cap	278.931	279.938	8	4	8	0	3	0
Screw Cap	424.029	425.036	19	17	2	3	4	0
Screw Cap	267.018	268.025	12	8	8	0	1	0
Screw Cap	284.018	285.025	11	8	8	1	0	0
Screw Cap	279.997	281.004	7	9	2	5	2	0
Screw Cap	481.045	482.052	18	20	12	0	1	0
Screw Cap	366.119	367.127	21	17	8	1	0	0
Screw Cap	405.039	406.046	18	9	10	4	2	0
Screw Cap	334.006	335.013	13	11	7	1	2	0

Screw Cap	248.971	249.978	6	7	8	0	1	0
Screw Cap	328.042	329.050	11	11	9	3	0	0
Screw Cap	315.058	316.065	20	10	7	0	2	0
Screw Cap	387.039	388.046	16	15	10	0	1	0
Screw Cap	255.018	256.025	12	7	8	0	1	0
Screw Cap	208.976	209.983	6	5	7	0	1	0
Screw Cap	254.982	255.989	8	6	9	0	1	0
Screw Cap	202.965	203.973	4	6	6	0	1	0
Screw Cap	210.974	211.981	8	5	5	0	2	0
Screw Cap	309.101	310.109	22	12	7	0	1	0
Screw Cap	279.018	280.025	12	9	8	0	1	0
Screw Cap	307.049	308.057	16	11	8	0	1	0
Screw Cap	369.029	370.036	14	15	9	0	1	0
Screw Cap	370.032	371.039	17	9	5	5	3	0
Screw Cap	170.997	172.004	8	3	6	0	1	0
Screw Cap	289.072	290.079	14	15	6	0	0	0
Screw Cap	379.103	380.111	20	18	9	0	0	0
Screw Cap	353.128	354.135	26	14	8	0	1	0
Screw Cap	228.985	229.992	10	5	6	0	2	0
Screw Cap	280.021	281.028	7	10	7	3	0	0
Screw Cap	489.086	490.093	22	23	10	0	1	0
Screw Cap	274.987	275.994	8	9	8	0	1	0
Screw Cap	154.966	155.973	4	2	6	0	1	0
Screw Cap	311.008	312.015	12	9	10	0	1	0
Screw Cap	474.065	475.072	21	17	9	3	2	0
Screw Cap	245.139	246.147	22	12	5	0	0	0
Screw Cap	204.981	205.989	6	6	6	0	1	0
Screw Cap	213.007	214.015	10	5	7	0	1	0
Screw Cap	282.011	283.018	13	8	6	1	2	0
Screw Cap	355.091	356.098	21	11	9	2	0	1
Screw Cap	379.029	380.036	20	13	3	2	4	0
Screw Cap	214.969	215.976	8	4	6	0	2	0
Screw Cap	397.071	398.078	18	16	8	2	1	0
Screw Cap	389.040	390.047	18	11	13	0	1	0
Screw Cap	388.980	389.987	14	13	0	4	4	0
Screw Cap	210.008	211.015	9	5	6	1	1	0
Screw Cap	292.072	293.080	20	8	6	1	0	1
Screw Cap	279.127	280.134	24	12	5	0	1	0
Screw Cap	331.053	332.060	20	10	8	0	2	0
Screw Cap	227.129	228.136	20	12	4	0	0	0
Screw Cap	360.050	361.058	12	14	5	5	0	1
Screw Cap	276.120	277.127	19	10	6	3	0	0
Screw Cap	398.079	399.087	22	12	8	3	1	1
Screw Cap	401.066	402.073	18	15	9	2	1	0
Screw Cap	222.938	223.945	4	5	6	0	2	0
Screw Cap	336.059	337.066	11	12	7	5	0	0
Screw Cap	363.087	364.094	20	13	8	2	1	0
Screw Cap	335.055	336.063	16	11	8	2	1	0
Screw Cap	218.946	219.953	8	3	5	0	3	0
Screw Cap	233.032	234.039	6	9	4	4	0	0

Screw Cap	265.980	266.987	9	7	6	1	2	0
Screw Cap	217.995	219.002	9	7	3	1	2	0
Screw Cap	193.029	194.036	10	5	4	2	1	0
Screw Cap	291.029	292.037	12	9	7	2	1	0
Screw Cap	387.049	388.056	17	15	10	0	0	1
Screw Cap	252.985	253.992	10	7	6	0	2	0
Screw Cap	272.974	273.982	10	6	8	0	2	0
Screw Cap	212.953	213.961	6	4	6	0	2	0
Screw Cap	339.021	340.029	16	11	8	0	2	0
Screw Cap	224.953	225.961	6	5	6	0	2	0
Screw Cap	309.011	310.018	14	10	7	0	2	0
Screw Cap	270.995	272.002	12	7	7	0	2	0
Screw Cap	232.995	234.002	10	8	4	0	2	0
Screw Cap	242.964	243.971	8	5	7	0	2	0
Screw Cap	369.061	370.068	18	11	10	2	1	0
Screw Cap	469.104	470.111	22	18	9	4	1	0
Screw Cap	375.060	376.068	20	11	12	0	1	0
Screw Cap	269.034	270.041	14	8	8	0	1	0
Screw Cap	252.992	253.999	10	12	0	0	2	0
Screw Cap	276.969	277.977	10	5	9	0	2	0
Screw Cap	345.119	346.126	22	15	9	0	0	0
Screw Cap	365.996	367.003	13	11	9	1	2	0
Screw Cap	308.992	309.999	10	9	10	0	1	0
Screw Cap	305.070	306.077	18	12	7	0	1	0
Screw Cap	335.081	336.088	20	13	8	0	1	0
Screw Cap	236.060	237.067	15	8	5	1	1	0
Screw Cap	196.055	197.062	11	8	1	3	1	0
Screw Cap	438.100	439.108	22	17	5	5	1	1
Screw Cap	357.050	358.057	18	11	11	0	1	0
Screw Cap	247.028	248.035	12	9	6	0	1	0
Screw Cap	296.008	297.015	11	8	9	1	1	0
Screw Cap	282.029	283.036	13	8	8	1	1	0
Screw Cap	240.026	241.033	7	8	6	3	0	0
Screw Cap	266.982	267.989	8	7	9	0	1	0
Screw Cap	491.162	492.169	32	17	16	0	0	0
Screw Cap	297.011	298.018	14	9	7	0	2	0
Screw Cap	286.990	287.997	12	7	8	0	2	0
Screw Cap	312.989	313.996	11	9	8	0	0	1
Screw Cap	362.055	363.062	17	13	9	1	1	0
Screw Cap	255.996	257.003	11	6	6	1	2	0
Screw Cap	290.075	291.082	17	9	2	5	2	0
Screw Cap	347.029	348.036	16	9	12	0	1	0
Screw Cap	243,000	244.008	12	6	6	0	2	0
Screw Cap	345.013	346.021	14	9	12	0	1	0
Screw Cap	303.054	304.062	16	12	7	0	1	0
Screw Cap	367.107	368.114	24	14	9	0	1	0
Screw Cap	303.091	304.098	20	13	6	0	1	0
Screw Cap	268.979	269.987	10	7	7	0	2	0
Screw Cap	412.049	413.056	19	12	9	3	2	0
Screw Cap	345.050	346.057	18	10	11	0	1	0

Screw Cap	305.055	306.062	18	8	10	0	1	0
Screw Cap	333.138	334.145	26	15	6	0	1	0
Screw Cap	280.013	281.021	11	8	8	1	1	0
Screw Cap	236.953	237.961	6	6	6	0	2	0
Screw Cap	279,000	280.008	12	9	6	0	2	0
Screw Cap	365.164	366.171	30	16	7	0	1	0
Screw Cap	207.008	208.015	8	5	5	2	1	0
Screw Cap	295.990	296.998	11	8	7	1	2	0
Screw Cap	264.002	265.009	7	9	1	5	2	0
Screw Cap	371.029	372.036	16	11	12	0	1	0
Screw Cap	373.045	374.052	18	11	12	0	1	0
Screw Cap	238.039	239.046	13	7	6	1	1	0
Screw Cap	477.125	478.132	26	19	14	0	0	0
Screw Cap	176.986	177.994	6	5	5	0	1	0
Screw Cap	201.023	202.030	10	8	4	0	1	0
Screw Cap	280.980	281.987	10	8	7	0	2	0
Screw Cap	206.997	208.004	8	6	6	0	1	0
Screw Cap	230.997	232.004	8	8	6	0	1	0
Screw Cap	361.045	362.052	18	10	12	0	1	0
Screw Cap	267.007	268.015	12	13	0	0	2	0
Screw Cap	190.966	191.973	4	5	6	0	1	0
Screw Cap	427.128	428.135	28	16	11	0	1	0
Screw Cap	232.028	233.036	11	8	5	1	1	0
Screw Cap	193.017	194.025	10	6	5	0	1	0
Screw Cap	258.959	259.966	8	5	8	0	2	0
Screw Cap	249.044	250.051	14	9	6	0	1	0
Screw Cap	287.096	288.103	20	13	5	0	1	0
Screw Cap	217.018	218.025	10	8	5	0	1	0
Screw Cap	272.992	273.999	10	6	10	0	1	0
Screw Cap	175.025	176.032	8	6	6	0	0	0
Screw Cap	201.007	202.015	10	4	7	0	1	0
Screw Cap	209.012	210.020	10	6	6	0	1	0
Screw Cap	225.007	226.015	10	6	7	0	1	0
Screw Cap	227.023	228.030	12	6	7	0	1	0
Screw Cap	260.974	261.982	10	5	8	0	2	0
Screw Cap	179.002	180.009	8	5	5	0	1	0
Screw Cap	224.990	225.997	10	6	5	0	2	0
Screw Cap	194.997	196.004	8	5	6	0	1	0
Screw Cap	261.044	262.051	14	10	6	0	1	0
Screw Cap	293.070	294.077	18	11	7	0	1	0
Screw Cap	257.034	258.041	14	7	8	0	1	0
Screw Cap	210.992	211.999	8	5	7	0	1	0
Screw Cap	325.035	326.042	14	9	9	2	1	0
Screw Cap	382.081	383.089	21	13	10	1	1	0
Screw Cap	265.998	267.005	9	7	8	1	1	0
Screw Cap	266.034	267.041	13	8	7	1	1	0
Screw Cap	205.018	206.025	10	7	5	0	1	0
Screw Cap	252.018	253.026	11	7	7	1	1	0
Screw Cap	239.023	240.030	12	7	7	0	1	0
Screw Cap	208.028	209.036	11	6	5	1	1	0

Screw Cap	377.039	378.047	18	10	13	0	1	0
Screw Cap	371.065	372.073	20	12	11	0	1	0
Screw Cap	290.039	291.046	18	8	4	1	1	1
Screw Cap	315.980	316.988	11	7	9	1	2	0
Screw Cap	286.954	287.961	8	6	9	0	2	0
Screw Cap	317.143	318.150	26	15	5	0	1	0
Screw Cap	343.089	344.096	24	12	7	0	2	0
Screw Cap	451.164	452.172	32	19	10	0	1	0
Screw Cap	285.029	286.036	14	8	9	0	1	0
Screw Cap	256.997	258.005	10	6	9	0	1	0
Screw Cap	254.964	255.971	8	6	7	0	2	0
Screw Cap	238.987	239.994	8	6	8	0	1	0
Screw Cap	243.018	244.025	12	6	8	0	1	0
Screw Cap	401.058	402.065	22	13	10	0	2	0
Screw Cap	294.013	295.020	9	10	2	5	2	0
Screw Cap	265.075	266.082	18	10	6	0	1	0
Screw Cap	281.034	282.041	14	9	8	0	1	0
Screw Cap	299.044	300.052	16	9	9	0	1	0
Screw Cap	300.969	301.977	10	7	9	0	2	0
Screw Cap	452.168	453.175	27	20	9	3	0	0
Screw Cap	229.002	230.010	10	5	8	0	1	0
Screw Cap	337.133	338.140	26	14	7	0	1	0
Screw Cap	164.986	165.994	6	4	5	0	1	0
Screw Cap	182.997	184.004	8	4	6	0	1	0
Screw Cap	197.049	198.056	14	6	5	0	1	0
Screw Cap	222.992	223.999	8	6	7	0	1	0
Screw Cap	241.039	242.046	14	7	7	0	1	0
Screw Cap	198.974	199.981	8	4	5	0	2	0
Screw Cap	157.014	158.022	6	6	5	0	0	0
Screw Cap	196.958	197.966	6	4	5	0	2	0
Screw Cap	241.002	242.010	10	6	8	0	1	0
Screw Cap	283.013	284.020	12	8	9	0	1	0
Screw Cap	342.998	344.005	12	9	12	0	1	0
Screw Cap	152.986	153.994	6	3	5	0	1	0
Screw Cap	192.981	193.989	6	5	6	0	1	0
Synthetic cork	287.060	288.068	20	13	1	0	3	0
Synthetic cork	315.083	316.091	16	12	8	2	0	0
Synthetic cork	297.073	298.080	14	12	7	2	0	0
Synthetic cork	343.078	344.086	16	13	9	2	0	0
Synthetic cork	352.064	353.071	23	14	1	1	3	0
Synthetic cork	243.051	244.058	12	10	7	0	0	0
Synthetic cork	223.025	224.032	8	10	6	0	0	0
Synthetic cork	425.097	426.104	26	12	14	0	1	0
Synthetic cork	285.098	286.105	18	13	7	0	0	0
Synthetic cork	313.035	314.042	14	8	9	2	1	0
Synthetic cork	262.969	263.976	8	8	6	0	2	0
Synthetic cork	245.030	246.038	10	9	8	0	0	0
Synthetic cork	287.054	288.061	17	8	9	0	0	1
Synthetic cork	398.088	399.095	21	12	10	3	1	0
Synthetic cork	199.028	200.035	12	5	6	0	1	0

Synthetic cork	254.067	255.074	13	11	6	1	0	0
Synthetic cork	415.060	416.067	17	13	6	6	1	1
Synthetic cork	355.162	356.170	24	15	6	4	0	0
Synthetic cork	229.021	230.028	14	6	5	0	2	0
Synthetic cork	325.023	326.031	14	10	10	0	1	0
Synthetic cork	201.993	203,000	5	5	4	3	1	0
Synthetic cork	186.974	187.981	8	3	5	0	2	0
Synthetic cork	200.990	201.997	10	4	5	0	2	0
Synthetic cork	296.002	297.009	11	7	6	3	2	0
Synthetic cork	259.072	260.080	12	13	4	2	0	0
Synthetic cork	321.029	322.036	14	11	9	0	1	0
Synthetic cork	511.098	512.105	28	15	17	0	1	0
Synthetic cork	465.004	466.012	19	11	12	2	2	1
Synthetic cork	451.025	452.032	21	11	11	2	2	1
Synthetic cork	377.051	378.059	19	12	4	4	2	1
Synthetic cork	412.955	413.963	15	7	10	2	3	1
Synthetic cork	386.157	387.164	25	16	8	3	0	0
Synthetic cork	163.061	164.068	12	6	5	0	0	0
Synthetic cork	302.949	303.956	8	6	10	0	2	0
Synthetic cork	296.992	297.999	10	8	10	0	1	0
Synthetic cork	297.029	298.036	14	9	9	0	1	0
Synthetic cork	196.061	197.069	11	9	4	1	0	0
Synthetic cork	206.979	207.986	8	6	4	0	2	0
Synthetic cork	426.020	427.027	18	12	8	3	2	1
Synthetic cork	219.992	220.999	7	6	6	1	1	0
Synthetic cork	389.025	390.032	19	10	8	2	2	1
Synthetic cork	396.960	397.968	15	7	9	2	3	1
Synthetic cork	319.067	320.074	16	12	10	0	0	0
Synthetic cork	360.006	361.014	15	9	10	1	2	0
Synthetic cork	465.102	466.109	27	14	15	0	0	1
Synthetic cork	388.012	389.020	12	11	7	5	1	1
Synthetic cork	157.051	158.058	10	7	4	0	0	0
Synthetic cork	131.035	132.042	8	5	4	0	0	0
Synthetic cork	340.050	341.057	15	14	7	1	1	0
Synthetic cork	191.020	192.027	8	6	7	0	0	0
Synthetic cork	326.060	327.067	17	8	5	5	2	0
Synthetic cork	383.119	384.127	24	14	12	0	0	0
Synthetic cork	295.103	296.111	20	11	9	0	0	0
Synthetic cork	237.062	238.069	14	8	8	0	0	0
Synthetic cork	312.065	313.073	19	15	0	1	2	0
Synthetic cork	187.134	188.141	20	10	3	0	0	0
Synthetic cork	428.050	429.058	19	13	13	1	1	0
Synthetic cork	270.011	271.018	13	7	6	1	2	0
Synthetic cork	268.991	269.998	10	6	6	2	2	0
Synthetic cork	302.055	303.062	17	8	9	1	1	0
Synthetic cork	217.072	218.079	14	9	6	0	0	0
Synthetic cork	557.100	558.107	27	22	11	2	1	1
Synthetic cork	185.082	186.089	14	9	4	0	0	0
Synthetic cork	506.961	507.968	16	13	11	2	3	0
Synthetic cork	441.092	442.099	26	12	15	0	1	0

Synthetic cork	368.058	369.066	16	13	4	5	1	1
Synthetic cork	433.031	434.038	19	13	12	0	0	1
Synthetic cork	287.023	288.030	12	11	7	0	1	0
Synthetic cork	323.985	324.993	11	9	8	1	2	0
Synthetic cork	256.083	257.090	15	11	6	1	0	0
Synthetic cork	384.024	385.032	15	11	12	1	1	0
Synthetic cork	244.997	246.005	10	5	9	0	1	0
Synthetic cork	411.081	412.089	24	11	14	0	1	0
Synthetic cork	293.007	294.014	10	10	8	0	0	0
Synthetic cork	374.022	375.029	17	10	10	1	2	0
Synthetic cork	294.022	295.029	17	11	0	1	3	0
Synthetic cork	185.045	186.053	10	8	5	0	0	0
Synthetic cork	293.018	294.026	14	6	11	0	1	0
Synthetic cork	225.040	226.048	10	10	6	0	0	0
Synthetic cork	277.093	278.100	18	11	8	0	0	0
Synthetic cork	267.072	268.079	16	9	9	0	0	0
Synthetic cork	241.108	242.115	18	12	5	0	0	0
Synthetic cork	130.999	132.006	4	4	5	0	0	0
Synthetic cork	184.061	185.069	11	8	4	1	0	0
Synthetic cork	227.056	228.063	12	10	6	0	0	0
Synthetic cork	245.067	246.074	14	10	7	0	0	0
Synthetic cork	349.060	350.067	18	13	9	0	1	0
Synthetic cork	202.108	203.116	17	9	4	1	0	0
Synthetic cork	346.071	347.078	14	14	4	5	0	1
Synthetic cork	575.042	576.049	17	17	15	6	0	1
Synthetic cork	238.072	239.079	13	11	5	1	0	0
Synthetic cork	256.046	257.054	11	10	7	1	0	0
Synthetic cork	269.975	270.982	9	6	7	1	2	0
Synthetic cork	285.062	286.069	14	12	8	0	0	0
Synthetic cork	517.178	518.185	34	19	16	0	0	0
Synthetic cork	199.025	200.032	8	8	6	0	0	0
Synthetic cork	296.045	297.052	15	9	8	1	1	0
Synthetic cork	264.985	265.992	10	8	6	0	2	0
Synthetic cork	320.099	321.106	19	12	9	1	0	0
Synthetic cork	338.073	339.080	17	11	11	1	0	0
Synthetic cork	347.989	348.996	15	8	8	1	3	0
Synthetic cork	451.022	452.029	17	14	11	2	1	1
Synthetic cork	261.017	262.024	10	10	6	0	0	0
Synthetic cork	224.056	225.064	11	10	5	1	0	0
Synthetic cork	301.114	302.121	22	10	10	0	0	0
Synthetic cork	288.072	289.080	15	11	8	1	0	0
Synthetic cork	280.104	281.111	19	10	8	1	0	0
Synthetic cork	402.133	403.141	22	18	4	5	0	1
Synthetic cork	407.131	408.138	24	15	11	2	0	0
Synthetic cork	182.046	183.053	9	8	4	1	0	0
Synthetic cork	268.028	269.036	11	11	5	1	1	0
Synthetic cork	246.992	247.999	8	8	7	0	1	0
Synthetic cork	252.088	253.095	15	12	5	1	0	0
Synthetic cork	274.057	275.064	13	10	8	1	0	0
Synthetic cork	213.040	214.048	10	9	6	0	0	0

Synthetic cork	214.072	215.079	13	9	5	1	0	0
Synthetic cork	343.088	344.095	20	11	12	0	0	0
Synthetic cork	351.060	352.068	20	9	12	0	1	0
Synthetic cork	369.039	370.046	18	17	3	0	2	0
Synthetic cork	188.056	189.064	11	7	5	1	0	0
Synthetic cork	312.016	313.023	15	6	9	1	1	0
Synthetic cork	355.082	356.089	20	11	9	2	1	0
Synthetic cork	279.039	280.046	12	9	6	2	0	0
Synthetic cork	281.035	282.042	10	10	4	4	1	0
Synthetic cork	261.062	262.069	14	10	8	0	0	0
Synthetic cork	251.114	252.121	20	10	7	0	0	0
Synthetic cork	397.099	398.106	22	14	13	0	0	0
Synthetic cork	242.103	243.111	17	11	5	1	0	0
Synthetic cork	247.046	248.053	12	9	8	0	0	0
Synthetic cork	383.083	384.090	20	13	13	0	0	0
Synthetic cork	323.098	324.106	20	12	10	0	0	0
Synthetic cork	271.103	272.111	20	9	9	0	0	0
Synthetic cork	204.088	205.095	15	8	5	1	0	0
Synthetic cork	206.046	207.053	9	10	4	1	0	0
Synthetic cork	456.172	457.180	31	17	13	1	0	0
Synthetic cork	327.130	328.137	24	12	10	0	0	0
Synthetic cork	229.035	230.043	10	9	7	0	0	0
Synthetic cork	271.072	272.079	16	6	6	4	1	0
Synthetic cork	273.109	274.116	18	11	6	2	0	0
Synthetic cork	314.088	315.095	17	13	8	1	0	0
Synthetic cork	372.018	373.025	15	9	9	3	2	0
Synthetic cork	204.967	205.974	6	6	0	2	2	0
Synthetic cork	287.041	288.048	12	11	9	0	0	0
Synthetic cork	260.974	261.981	7	7	5	2	1	1
Synthetic cork	409.997	411.004	13	11	10	3	2	0
Synthetic cork	533.172	534.180	34	19	17	0	0	0
Synthetic cork	251.041	252.048	12	8	9	0	0	0
Synthetic cork	281.067	282.074	14	13	7	0	0	0
Synthetic cork	361.056	362.064	14	17	9	0	0	0
Synthetic cork	228.966	229.973	6	4	9	0	1	0
Synthetic cork	404.005	405.012	15	13	6	3	3	0
Synthetic cork	331.041	332.048	17	13	6	0	1	1
Synthetic cork	447.997	449.004	12	12	10	5	1	1
Synthetic cork	320.019	321.026	8	11	5	5	0	1
Synthetic cork	155.035	156.042	8	7	4	0	0	0
Synthetic cork	507.015	508.022	21	13	13	2	2	1
Synthetic cork	466.005	467.013	17	14	9	3	3	0
Synthetic cork	463.992	464.999	12	12	11	5	1	1
Synthetic cork	263.026	264.033	13	8	4	2	1	1
Synthetic cork	416.983	417.991	15	10	10	2	2	1
Synthetic cork	460.973	461.980	15	11	12	2	2	1
Synthetic cork	489.004	490.012	19	13	12	2	2	1
Synthetic cork	266.967	267.974	9	6	4	2	2	1
Synthetic cork	319.016	320.023	13	10	6	2	1	1
Synthetic cork	470.979	471.986	16	10	13	2	2	0

Synthetic cork	296.977	297.985	11	7	5	2	2	1
Synthetic cork	463.989	464.997	15	14	9	3	3	0
Synthetic cork	391.948	392.956	8	11	11	1	1	1
Synthetic cork	475.025	476.032	21	13	11	2	2	1
Synthetic cork	488.951	489.958	14	13	10	2	3	0
Synthetic cork	214.987	215.994	8	4	8	0	1	0
Synthetic cork	389.006	390.014	14	10	10	2	1	0
Synthetic cork	305.161	306.168	26	14	7	0	0	0
Synthetic cork	300.989	301.996	11	8	8	0	0	1
Synthetic cork	352.019	353.027	16	10	5	3	2	1
Synthetic cork	255.051	256.058	12	11	7	0	0	0
Synthetic cork	409.029	410.037	18	10	15	0	1	0
Synthetic cork	439.076	440.084	24	12	15	0	1	0
Synthetic cork	440.080	441.087	20	16	6	5	1	1
Synthetic cork	441.072	442.079	18	16	9	4	1	0
Synthetic cork	441.081	442.088	22	16	12	0	0	0
Synthetic cork	368.911	369.918	6	5	11	2	2	0
Synthetic cork	462.989	463.996	17	11	12	2	2	1
Synthetic cork	446.994	448.001	17	11	11	2	2	1
Synthetic cork	419.966	420.973	8	10	10	5	1	1
Synthetic cork	270.942	271.949	5	6	8	0	0	1
Synthetic cork	418.962	419.970	13	9	11	2	2	1
Synthetic cork	423.045	424.052	20	11	15	0	1	0
Synthetic cork	270.943	271.951	4	5	7	2	1	0
Synthetic cork	419.963	420.971	11	12	8	3	3	0
Synthetic cork	312.972	313.980	11	7	6	2	2	1
Synthetic cork	384.009	385.017	16	10	7	3	2	1
Synthetic cork	338.988	339.995	13	9	6	2	2	1
Synthetic cork	355.019	356.027	17	10	6	2	2	1
Synthetic cork	387.009	388.016	17	10	8	2	2	1
Synthetic cork	268.946	269.953	7	5	5	2	2	1
Synthetic cork	301.009	302.016	15	7	5	2	2	1
Synthetic cork	310.957	311.964	9	7	6	2	2	1
Synthetic cork	312.954	313.961	6	7	8	2	1	0
Synthetic cork	244.119	245.126	19	11	5	1	0	0
Synthetic cork	267.015	268.022	8	11	8	0	0	0
Synthetic cork	506.998	508.005	20	14	10	2	3	0
Synthetic cork	220.083	221.090	15	8	6	1	0	0
Synthetic cork	257.067	258.074	14	11	7	0	0	0
Synthetic cork	171.030	172.037	8	7	5	0	0	0
Synthetic cork	448.991	449.998	14	11	13	2	1	0
Synthetic cork	183.033	184.041	12	5	5	0	1	0
Synthetic cork	331.070	332.078	20	10	10	0	1	0
Synthetic cork	347.030	348.037	20	10	7	0	3	0
Synthetic cork	388.977	389.984	12	12	7	2	1	2
Synthetic cork	407.050	408.057	20	11	14	0	1	0
Synthetic cork	298.956	299.964	9	6	6	2	2	1
Synthetic cork	236.093	237.100	15	12	4	1	0	0
Synthetic cork	483.154	484.161	32	19	12	0	1	0
Synthetic cork	321.013	322.021	14	7	12	0	1	0

Synthetic cork	601.130	602.137	35	21	12	2	2	1
Synthetic cork	392.033	393.040	19	10	11	1	2	0
Synthetic cork	611.063	612.070	29	17	16	2	2	1
Synthetic cork	303.018	304.025	12	11	8	0	1	0
Synthetic cork	373.078	374.085	18	15	11	0	0	0
Synthetic cork	432.030	433.037	19	14	3	5	4	0
Synthetic cork	572.122	573.129	28	21	10	5	1	1
Synthetic cork	333.014	334.021	18	9	7	0	3	0
Synthetic cork	401.109	402.116	22	17	11	0	0	0
Synthetic cork	329.055	330.062	18	10	10	0	1	0
Synthetic cork	478.966	479.973	16	12	10	2	3	0
Synthetic cork	435.943	436.950	11	8	12	3	3	0
Synthetic cork	434.940	435.947	12	10	9	2	3	0
Synthetic cork	341.030	342.037	14	9	10	2	1	0
Synthetic cork	318.050	319.057	17	8	10	1	1	0
Synthetic cork	307.034	308.041	16	7	11	0	1	0
Synthetic cork	225.080	226.087	18	8	5	0	1	0
Synthetic cork	288.987	289.994	10	6	11	0	1	0
Synthetic cork	429.020	430.027	18	13	8	2	2	0
Synthetic cork	430.999	432.006	17	11	10	2	2	1
Synthetic cork	287.008	288.015	12	7	10	0	1	0
Synthetic cork	253.111	254.119	22	10	5	0	1	0
Synthetic cork	211.064	212.072	16	7	5	0	1	0
Synthetic cork	212.068	213.075	11	8	4	3	0	0
Synthetic cork	253.075	254.082	18	9	6	0	1	0
Synthetic cork	257.030	258.038	10	10	8	0	0	0
Synthetic cork	279.003	280.011	12	9	2	2	2	0
Synthetic cork	300.029	301.036	15	13	1	1	2	0
Synthetic cork	476.951	477.958	14	12	10	2	3	0
Synthetic cork	191.056	192.063	12	7	6	0	0	0
Synthetic cork	325.078	326.085	18	11	11	0	0	0
Synthetic cork	480.982	481.989	18	12	10	2	3	0
Synthetic cork	571.119	572.126	33	20	11	2	2	1
Synthetic cork	423.060	424.068	20	15	12	0	1	0
Synthetic cork	226.963	227.970	5	4	5	2	0	1
Synthetic cork	390.998	392.005	12	13	12	0	1	0
Synthetic cork	431.027	432.034	17	12	11	2	0	1
Synthetic cork	356.945	357.952	10	9	5	2	3	0
Synthetic cork	338.048	339.055	21	13	1	1	3	0
Synthetic cork	337.045	338.052	18	8	12	0	1	0
Synthetic cork	291.003	292.010	12	6	11	0	1	0
Synthetic cork	292.006	293.013	15	11	0	1	3	0
Synthetic cork	367.055	368.062	20	9	13	0	1	0
Synthetic cork	292.999	294.006	11	10	6	0	0	1
Synthetic cork	217.035	218.043	10	8	7	0	0	0
Synthetic cork	248.981	249.988	6	8	7	0	0	0
Synthetic cork	183.030	184.037	8	8	5	0	0	0
Synthetic cork	385.154	386.161	30	15	9	0	1	0
Synthetic cork	216.930	217.938	6	3	5	0	3	0
Synthetic cork	431.044	432.051	20	12	11	2	2	0

Synthetic cork	357.122	358.130	26	13	9	0	1	0
Synthetic cork	366.029	367.036	13	15	8	1	1	0
Synthetic cork	302.070	303.078	17	12	6	1	1	0
Synthetic cork	232.925	233.933	6	3	6	0	3	0
Synthetic cork	260.957	261.964	10	5	6	0	3	0
Synthetic cork	510.956	511.963	17	11	13	2	3	1
Synthetic cork	229.962	230.969	9	4	4	1	3	0
Synthetic cork	303.963	304.970	11	6	7	1	3	0
Synthetic cork	231.941	232.949	7	3	5	1	3	0
Synthetic cork	283.006	284.014	12	7	6	2	2	0
Synthetic cork	216.967	217.974	10	4	4	0	3	0
Synthetic cork	289.947	290.954	9	5	7	1	3	0
Synthetic cork	333.973	334.980	13	7	8	1	3	0
Synthetic cork	389.052	390.059	22	11	7	2	3	0

FT-ICR-MS example spectra of wines treated with GSH after alcoholic fermentation (50AF) or at bottling (20B; 50B) and bottled with screw cap (C) or synthetic cork (S).

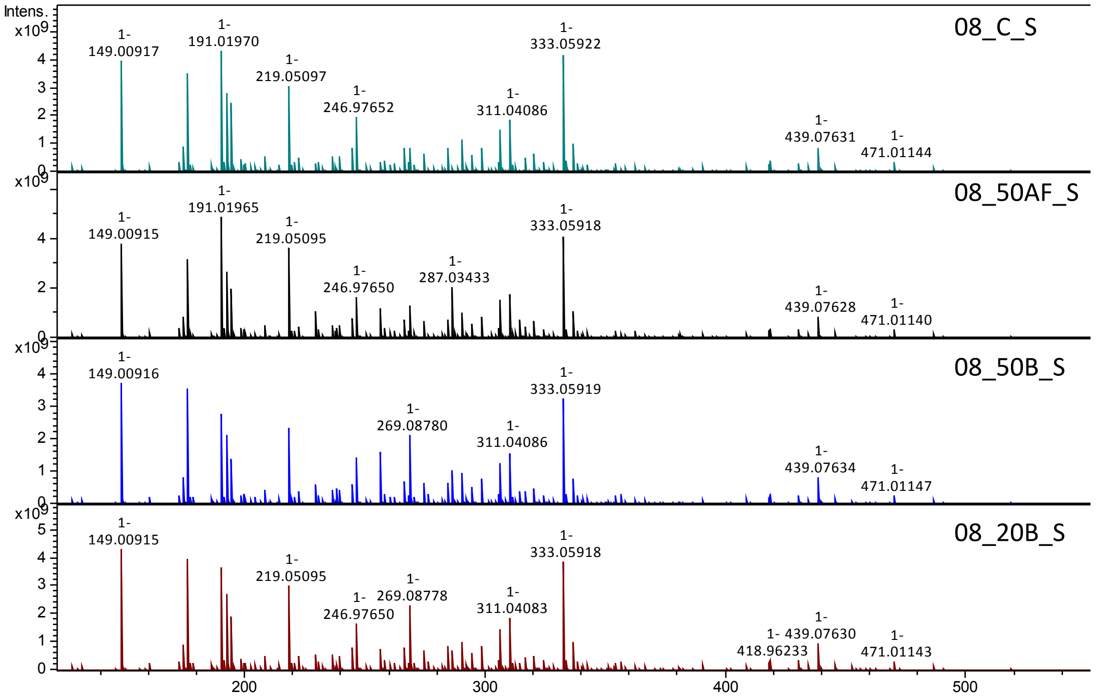

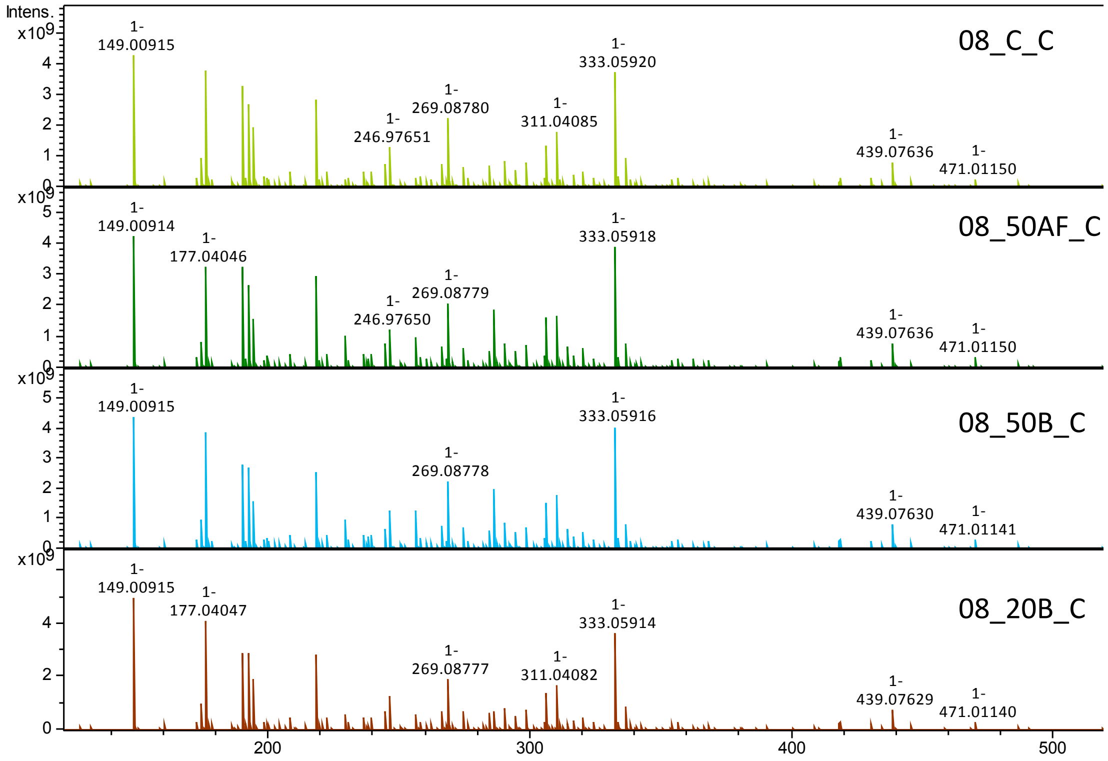

Figure S1: FT-ICR-MS spectrum of pyroglutamic acid

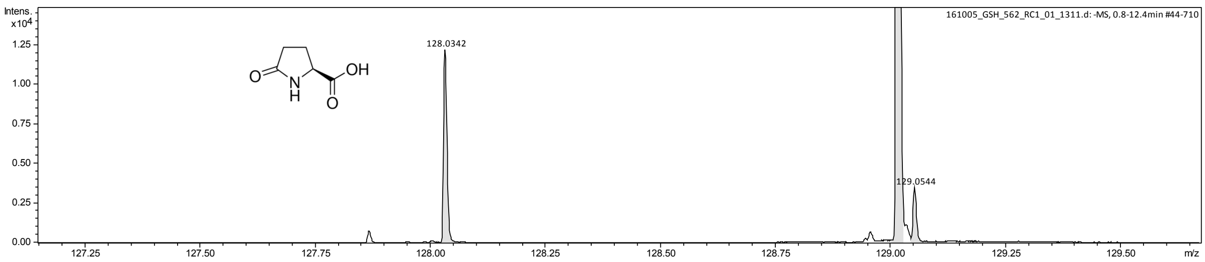

Figure S2: Ion m/z 128.03531 distributions putatively identified as pyroglutamic acid in individual wine samples from 2008 and 2009 treated with GSH after alcoholic fermentation (50AF) or at bottling (20B; 50B) and bottled with screw cap (C) or synthetic cork (S).

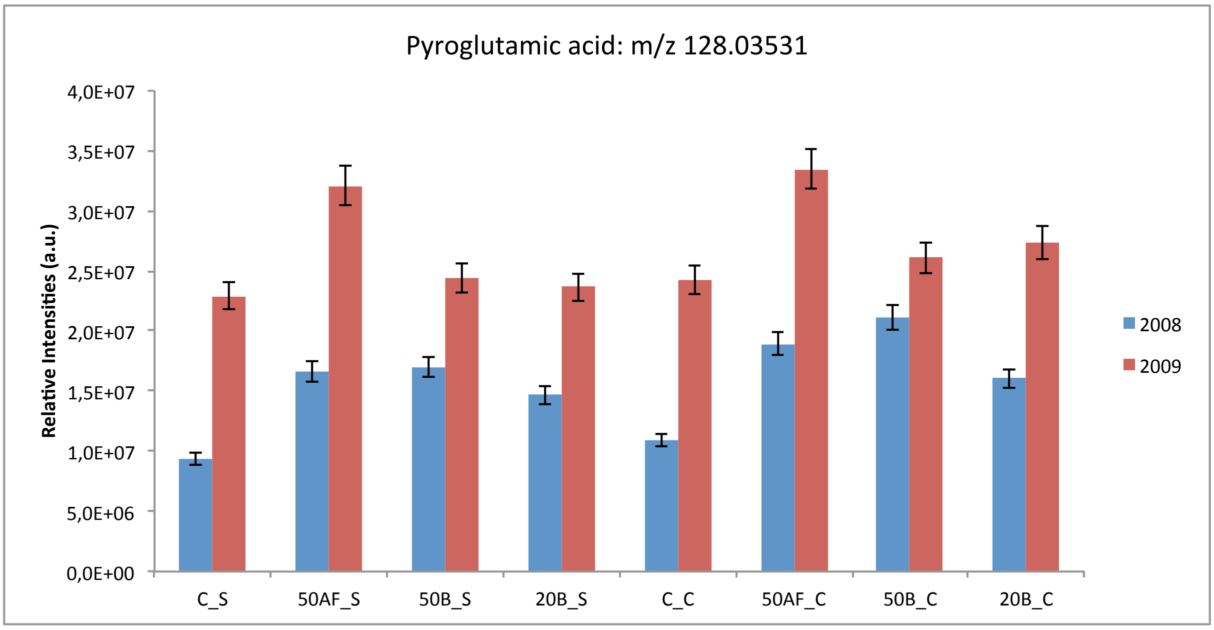

Figure S3: FT-ICR-MS spectrum of cys-S-sulfite

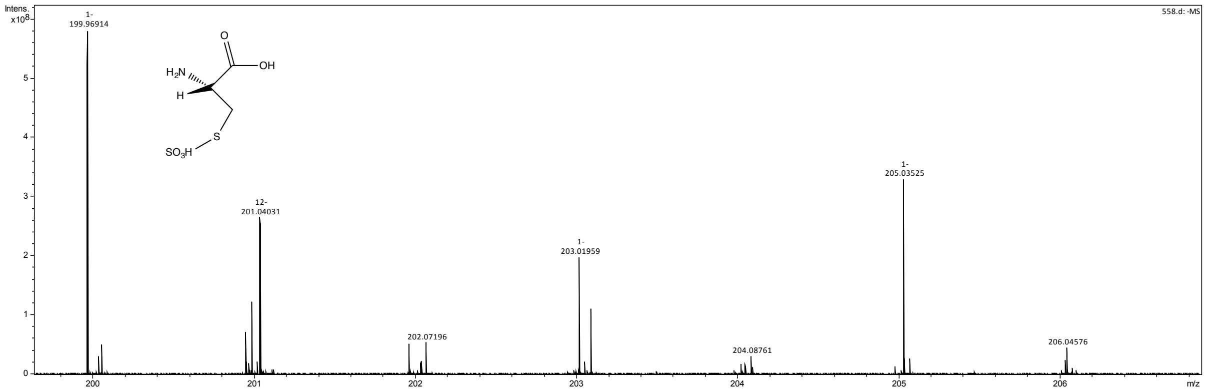

Figure S4: Ion m/z 199.96915 distributions putatively identified as cys-S-sulfite acid in individual wine samples from 2008 and 2009 treated with GSH after alcoholic fermentation (50AF) or at bottling (20B; 50B) and bottled with screw cap (C) or synthetic cork (S).

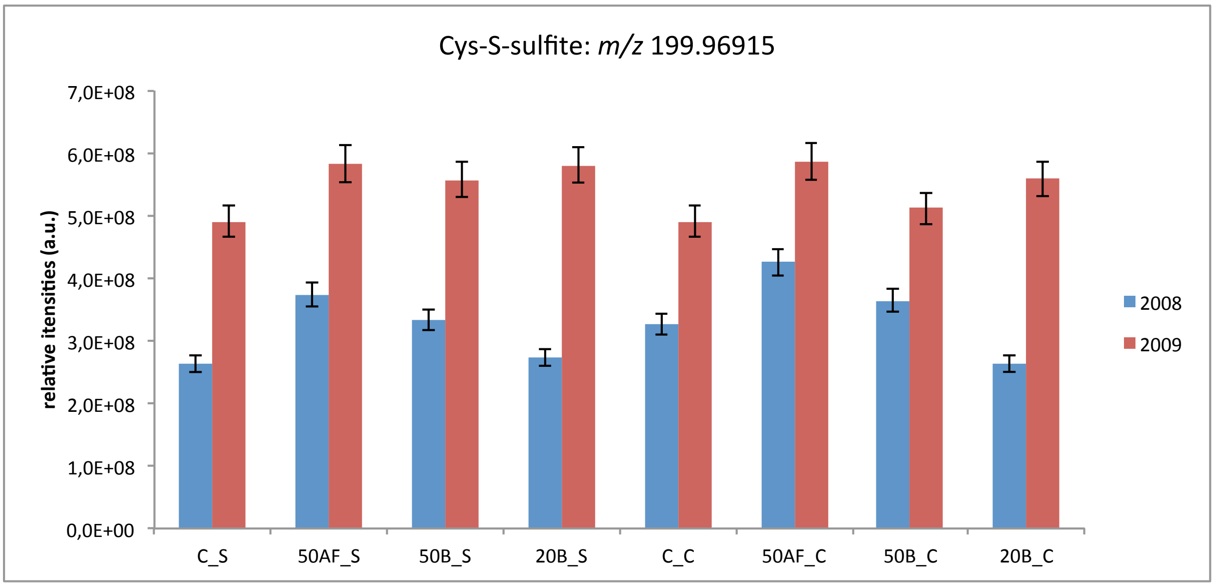

Figure S5: FT-ICR-MS spectrum of GSH-S-sulfite

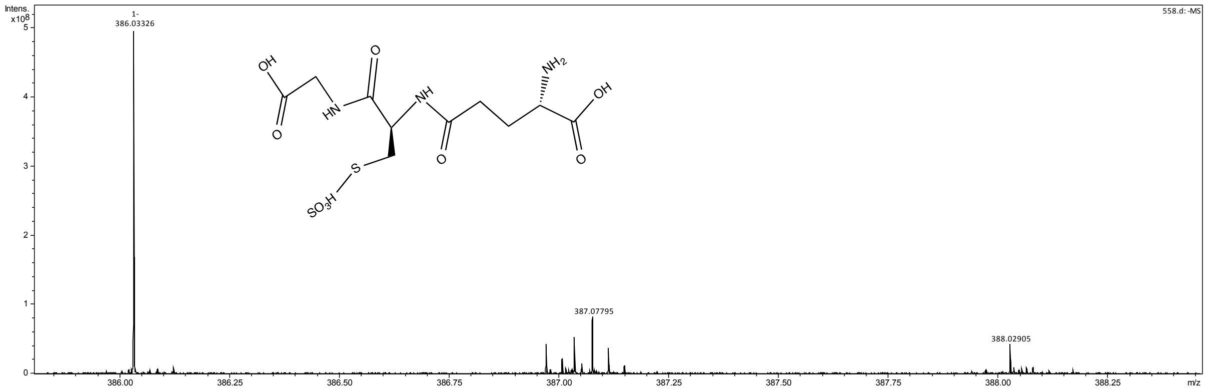

Figure S6: Ion m/z 386.03330 distributions putatively identified as GSH-S-sulfite acid in individual wine samples from 2008 and 2009 treated with GSH after alcoholic fermentation (50AF) or at bottling (20B; 50B) and bottled with screw cap (C) or synthetic cork (S).

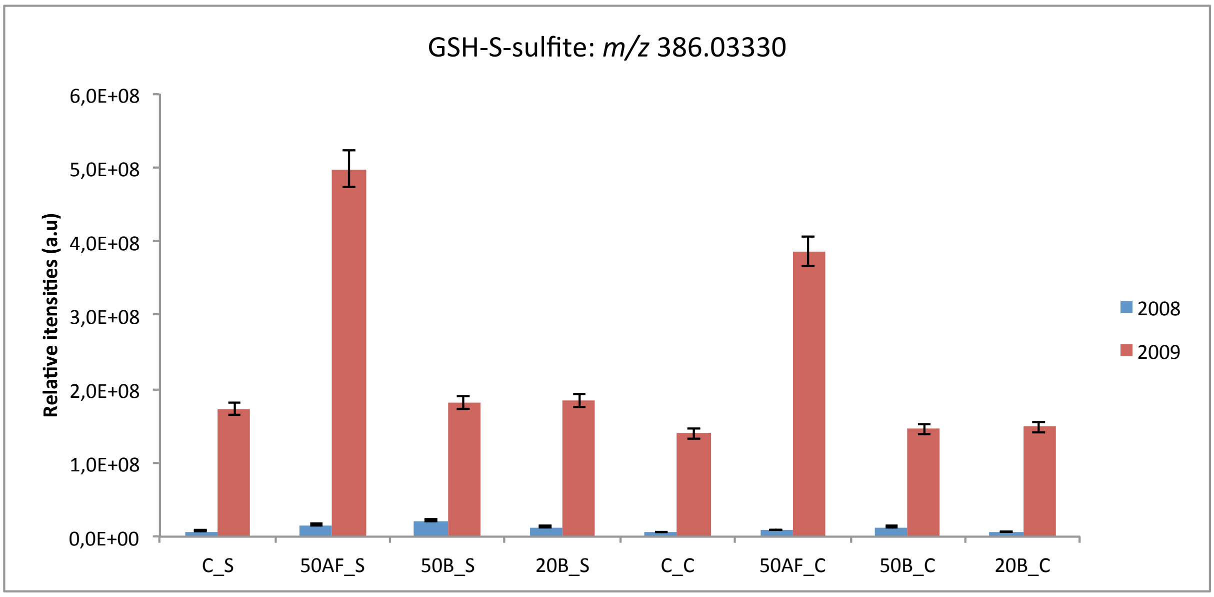

Figure S7: Ion intensities distribution of selected tentatively identified peptides in individual wine samples from 2008 treated with GSH after alcoholic fermentation (50AF) or at bottling (20B; 50B) and bottled with screw cap (C) or synthetic cork (S). ST ≤ 4%.

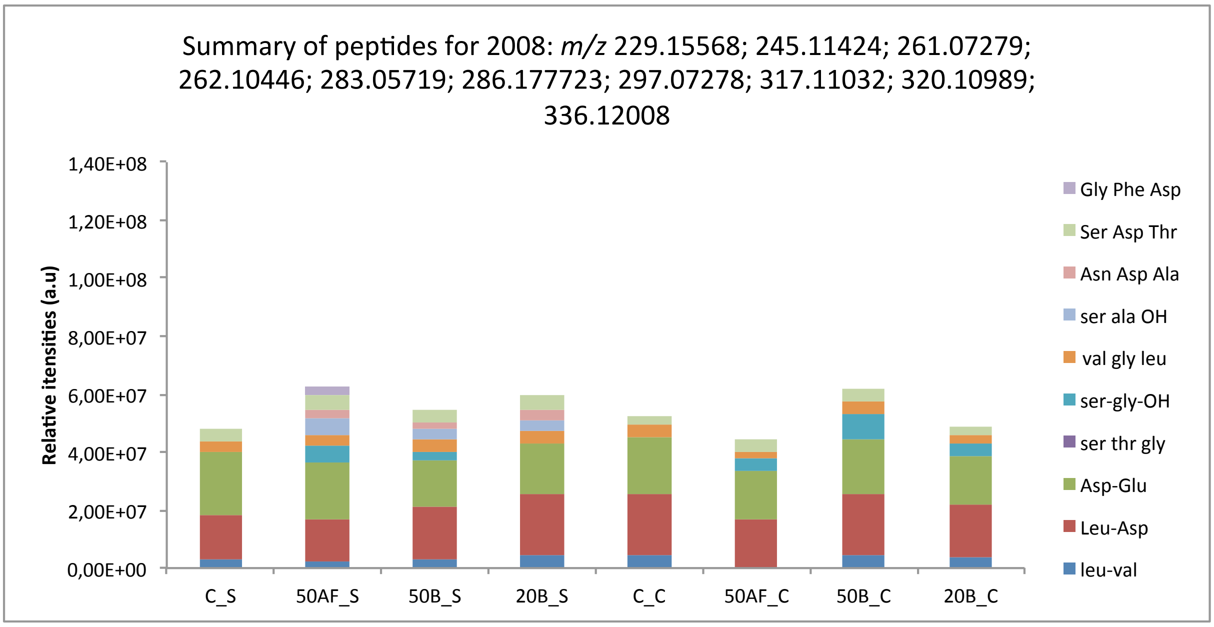

Figure S8: Ion intensities distribution of selected tentatively identified peptides in individual wine samples from 2009 treated with GSH after alcoholic fermentation (50AF) or at bottling (20B; 50B) and bottled with screw cap (C) or synthetic cork (S). ST ≤ 4%.

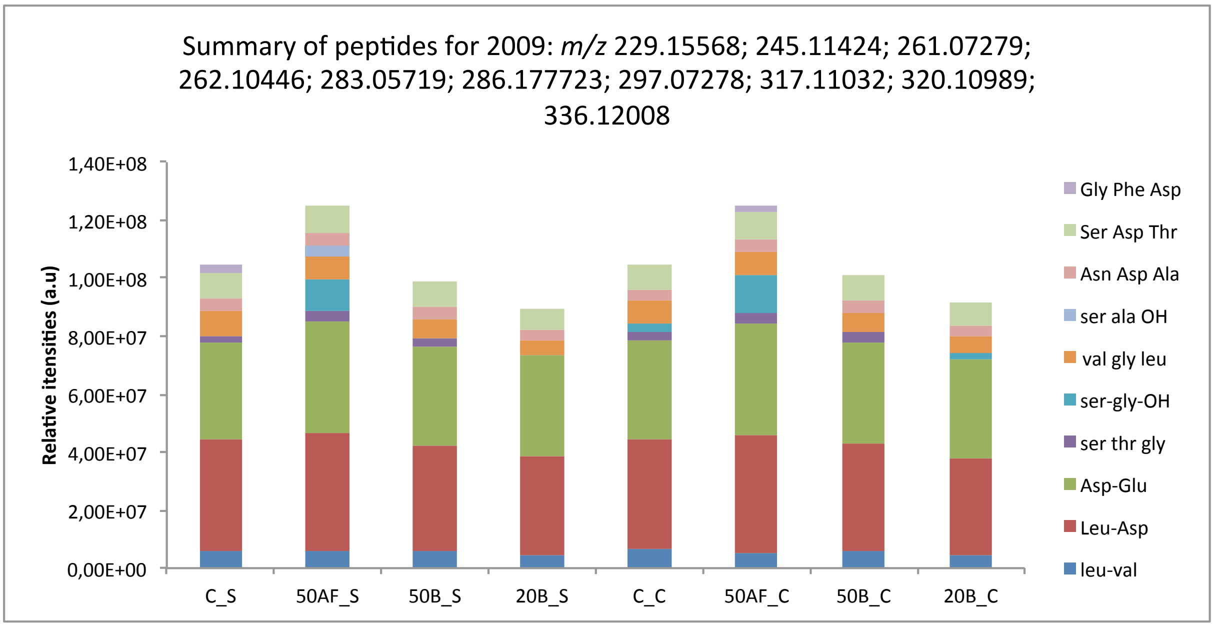

Figure S9: Ion intensities distribution of selected tentatively identified peptides in individual wine samples from 2009 treated with GSH after alcoholic fermentation (50AF) or at bottling (20B; 50B) and bottled with screw cap (C) or synthetic cork (S).

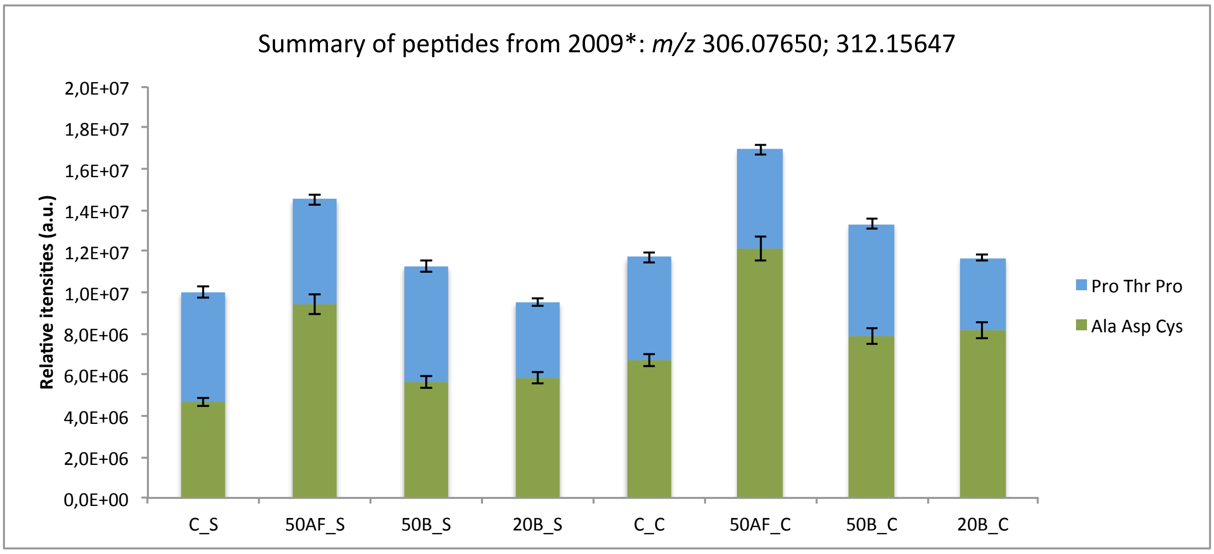

*ND in 2008 samples
